# Regional citrate anticoagulation versus systemic heparin anticoagulation for continuous kidney replacement therapy in intensive care

**DOI:** 10.1016/j.jcrc.2022.154218

**Published:** 2023-04

**Authors:** James C. Doidge, Doug W. Gould, Zia Sadique, Mark Borthwick, Robert A. Hatch, Fergus J. Caskey, Lui Forni, Robert F. Lawrence, Clare MacEwan, Marlies Ostermann, Paul R. Mouncey, David A. Harrison, Kathryn M. Rowan, J. Duncan Young, Peter J. Watkinson

**Affiliations:** a Intensive Care National Audit and Research Centre, 24 High Holborn, London WC1V 6AZ, United Kingdom; b Department of Health Services Research & Policy, London School of Hygiene & Tropical Medicine, London, United Kingdom; c Departments of Pharmacy and Critical Care, Oxford University Hospitals NHS Foundation Trust, Oxford OX3 9DU, United Kingdom; d Kadoorie Centre for Critical Care Research, Nuffield Department of Clinical Neurosciences, University of Oxford, OX3 9DU, United Kingdom; e Bristol Medical School, University of Bristol, 39 Whatley Road, Bristol BS8 2PS, United Kingdom; f UK Renal Registry, Brandon House, Building 20a1, Southmead Road, Bristol BS34 7RR, United Kingdom; g Department of Clinical and Experimental Medicine, Faculty of Health Sciences, University of Surrey, Guildford GU2 7XH, United Kingdom; h Intensive Care Unit, Royal Surrey County Hospital NHS Foundation Trust, Guildford GU2 7XX, United Kingdom; i Patient Representative, Address Withheld; j Oxford University Hospitals NHS Foundation Trust, John Radcliffe Hospital, Oxford OX3 9DU, United Kingdom; k King's College London, Guy's & St Thomas' Hospital, Department of Critical Care, Westminster Bridge Road, London SE1 7EH, United Kingdom; l Kadoorie Centre for Critical Care Research and Education, University of Oxford, John Radcliffe Hospital, Oxford OX3 9DU, United Kingdom; m Nuffield Department of Clinical Neurosciences, University of Oxford, Level 6, West Wing, John Radcliffe Hospital, Oxford OX3 9DU, United Kingdom; n NIHR Biomedical Research Centre, Oxford, Oxford University Hospitals NHS Trust, Kadoorie Centre for Critical Care Research and Education, Headley Way, Oxford OX3 9DU, United Kingdom

**Keywords:** Kidney replacement, Renal replacement, Anticoagulation, Citrate, Heparin, Intensive care

## Abstract

**Purpose:**

Many intensive care units (ICUs) have transitioned from systemic heparin anticoagulation (SHA) to regional citrate anticoagulation (RCA) for continuous kidney replacement therapy (CKRT). We evaluated the clinical and health economic impacts of ICU transition to RCA.

**Materials and methods:**

We surveyed all adult general ICUs in England and Wales to identify transition dates and conducted a micro-costing study in eight ICUs. We then conducted an interrupted time-series analysis of linked, routinely collected health records.

**Results:**

In 69,001 patients who received CKRT (8585 RCA, 60,416 SHA) in 181 ICUs between 2009 and 2017, transition to RCA was not associated with a change in 90-day mortality (adjusted odds ratio 0.98, 95% CI 0.89–1.08) but was associated with step-increases in duration of kidney support (0.53 days, 95% CI 0.28–0.79), advanced cardiovascular support (0.23 days, 95% CI 0.09–0.38) and ICU length of stay (0.86 days, 95% CI 0.24–1.49). The estimated one-year incremental net monetary benefit per patient was £ − 2376 (95% CI £ − 3841–£ − 911), with an estimated likelihood of cost-effectiveness of <0.1%.

**Conclusions:**

Transition to RCA was associated with significant increases in healthcare resource use, without corresponding clinical benefit, and is highly unlikely to be cost-effective over a one-year time horizon.

## Highlights

• No change in 90-day mortality with transition to regional citrate anticoagulation.
• Regional citrate anticoagulation increased intensive care stay and organ support.
• Regional citrate anticoagulation costs significantly more than systemic heparin.

## 1 Introduction

Approximately 10% of admissions to an intensive care unit (ICU) require kidney replacement therapy (KRT) [1]. Most ICUs use continuous kidney replacement therapy (CKRT), which often requires anticoagulation to prevent clotting in the extracorporeal circuit [2,3]. Heparin anticoagulation, is increasingly being replaced by regional citrate anticoagulation (RCA) for CKRT. With heparin anticoagulation (termed systemic heparin anticoagulation, SHA, in light of systemic anticoagulation effects even when infused into the kidney replacement circuit),clotting status is checked repeatedly to maintain therapeutic efficacy [4]. In contrast, RCA chelates calcium using a citrate solution added to blood entering the extracorporeal circuit, which is reversed by infusing calcium chloride or calcium gluconate as blood is returned to the circulation [5]. Risks associated with RCA include hypocalcaemia [6], hypophosphataemia [7,8], hypomagnesiaemia, alterations in the blood acid-base balance [5,9], and possibly increased infection rates [8].

Use of RCA in ICUs has increased since the 2012 KDIGO (Kidney Disease: Improving Global Outcomes) Clinical Practice Guideline for Acute Kidney Injury recommended, albeit weakly, RCA as first-line anticoagulation in CKRT [10]. However, adoption occurred without robust large-scale studies assessing patient-centered clinical outcomes and cost-effectiveness [6,8,[11], [12], [13]]. Prior to our study, systematic reviews of relatively small randomised controlled trials (RCTs) suggested no difference in mortality between the two methods, but prolonged filter life with RCA [6,11]. RCA was associated with lower risk of bleeding, but the clinical significance of bleeding was unclear. Very limited analysis of cost effectiveness had been undertaken [6].

In the United Kingdom, many ICUs have transitioned to RCA, allowing us to assess real-world effects in the population of patients treated throughout the healthcare system. This information provides a valuable complement to RCT findings, which can have limited generalisability. We linked routinely collected health records and pre-existing ICU research databases to evaluate the clinical and cost-effectiveness of transition to RCA in all adult, general ICUs in England and Wales.

## 2 Material and methods

### 2.1 Overview

We combined: (i) an ICU practice survey to determine dates of transition from SHA to RCA (ii) a micro-costing study to estimate treatment costs associated with SHA and RCA, and (iii) an interrupted time series analysis [14,15] of linked electronic health records to compare clinical outcomes for patients admitted before or after transition from SHA to RCA. Detailed methods can be found in the published protocol [16] and statistical analysis plan [17]. The study received approval from Oxford B Research Ethics Committee (REC reference: 18/SC/0204) and support under Section 251 of the NHS Act 2006 from the Confidentiality Advisory Group (CAG reference: 18/CAG/0070) and was registered at clinicaltrials.gov (NCT03545750).

### 2.2 Data sources: ICU practice survey

We surveyed all adult, general ICUs in England and Wales that were participating in the Case Mix Programme (CMP) national clinical audit of adult intensive care. Respondents provided information about if, and when, their ICU transitioned from SHA to RCA for CKRT, and, if applicable, any patient groups for whom ICUs that had commenced RCA continued to use SHA. See Supplementary Material for further detail.

### 2.3 Data sources: micro-costing study

To account for differences in filter life, we estimated the average number of CKRT sessions per calendar day, and CKRT hours per calendar day using anonymised patient data from the Post Intensive Care Risk-Adjusted Alerting and Monitoring (PICRAM) study of all patients treated on both Oxford general ICUs from 2009 to 2015 [18], and the Oxford ICU electronic clinical information system following PICRAM completion (2015–2017). These estimates were specifically constructed per calendar day, and not per linear day, for compatibility with data on duration of kidney support; however, because they include time not on CKRT during days of CKRT commencement and cessation, they cannot be interpreted as direct measures of filter life.

To estimate the staff time and consumables, we conducted a cognitive walk-through exercise with clinicians from seven ICUs (three SHA, four RCA). We combined outputs with published unit costs (see Supplementary Material for further detail).

### 2.4 Data sources: interrupted time-series analysis

We combined outputs from the ICU practice survey and micro-costing study with anonymised patient-level clinical data from the Case Mix Programme (CMP) national clinical audit of adult intensive care to identify the study population and hospital outcomes; Hospital Episode Statistics for England (HES) and Patient Episode Data for Wales (PEDW) to derive indicators of previous and subsequent hospitalisation; the UK Renal Registry (UKRR) to derive indicators of dialysis and kidney transplant; and Civil Registrations to derive indicators of mortality (Supplementary Table 3).

### 2.5 Patient selection

We included all patients aged at least 16 years who received CKRT between 1 April 2009 and 31 March 2017 in an ICU that participated in the practice survey. We excluded patients with end-stage kidney failure, liver failure, or kidney/multi-organ transplant; and patients admitted to standalone high dependency or specialist (e.g. cardiothoracic or neurosciences) ICUs.

### 2.6 Treatment

We identified receipt of RCA by admission to an ICU at least 6 months after the ICU commenced transition from SHA to RCA. We identified receipt of SHA by admission to an ICU prior to commencement of transition, and censored (excluded) patients admitted within the first 6 months after commencement of transition.

### 2.7 Clinical outcomes

Our primary clinical outcome was mortality at 90 days after ICU admission. We identified mortality from the CMP record where death occurred during hospital admission or from the linked Civil Registrations record where death occurred post-discharge. Our secondary clinical outcomes included 30–day and one-year mortality; calendar days of kidney support, advanced cardiovascular support, and advanced respiratory support (obtained from CMP record); ICU, subsequent hospital and total hospital length of stay (LOS; obtained from CMP record); bleeding during ICU admission, and thromboembolic episodes up to 90 days post-discharge (obtained from linked HES records); and end-stage kidney disease (ESKD) at 90 days (post-hoc) and one year (pre-specified) (obtained from linked UKRR records). See Supplementary Material, “Definitions and codes” for detailed definitions.

### 2.8 Health economic outcomes

Our primary economic outcome was one-year incremental net benefit (INB) and the associated likelihood of cost-effectiveness with a willingness-to-pay of £20,000 per quality-adjusted life year (QALY). Our secondary economic outcome was the extrapolated lifetime INB. We included costs of delivering the CKRT, costs of ICU and hospital length of stay, and costs of chronic kidney dialysis. We calculated the number of QALYs accrued by each patient in the first year after ICU admission using EuroQuol EQ − 5D − 3 L health-related quality of life (HRQoL) from the UK Intensive Care Outcome Network Study (ICON) study [19] for patients at three months and one year after ICU discharge. For patients who developed ESKD, we applied a decrement according to European norms (see Supplementary Material) [20].

### 2.9 Covariates

We selected patient covariates based on importance in established risk models for ICU [21]: age; comorbidities identified from previous hospital admission involving congestive cardiac failure, peripheral vascular disease, cerebrovascular disease, chronic pulmonary disease, chronic liver disease, or malignancy; recent history of haematological malignancy, severe immunocompromise, severe liver disease, metastatic disease, severe respiratory disease, and very severe cardiovascular disease; dependency prior to hospital admission; body mass index; location prior to admission to ICU; cardiopulmonary resuscitation within 24 h prior to ICU admission; primary reason for admission to ICU (body system); receipt of mechanical ventilation during first 24 h in ICU; and physiological recordings within first 24 h in ICU (see Supplementary Material for definitions and codes). We also included quarter of year to account for seasonal variation.

### 2.10 Statistical analysis

Because a lag in UKRR data collection limited follow-up for the ESKD outcome, we implemented a post hoc analysis of ESKD at 90 days and excluded patients with incomplete follow-up from analyses of ESKD only. Data sources and definitions ensured complete data for other outcomes. We imputed missing covariates using fully conditional specification implemented using the Multiple Imputation by Chained Equations (MICE) algorithm [22,23].

We implemented a multilevel interrupted time-series analysis, with interruption on the date of ICU transition from SHA to RCA, following the eight quality criteria described by Ramsay et al. [24] We used random effects multilevel generalised linear models to estimate the ICU-level effect of transitioning to RCA on patient-level outcomes. We report regression model results as the odds ratio or difference in means for the step-change and the odds ratio per year or difference in means per year for the change in trend. We estimated robust standard errors to allow for model misspecification and tested the joint significance of the step-change and change in trend.

We modelled health economic outcomes (costs and QALYs) using the same techniques as for clinical outcomes. We estimated INB at one year associated with a change from SHA to RCA valuing incremental QALYs according to a NICE recommended threshold willingness-to-pay of £20,000/QALY and subtracted incremental costs. Lifetime health economic outcomes were extrapolated by summarising the relative effects of RCA versus SHA on long-term costs and outcomes, as compared to age-gender matched general population.

Further detail on statistical models and assumptions is provided in Supplementary Material. All analysis was conducted using Stata MP 14 (StataCorp, Texas).

### 2.11 Subgroup analysis

We pre-specified a subgroup analysis of patients meeting Sepsis-3 criteria [25].

### 2.12 Sensitivity analyses

We pre-specified three sensitivity analyses: (i) including only ICUs with data from both before and after transition from SHA to RCA, (ii) a ‘best case’ scenario employing the highest treatment costs reported by any ICU in the cognitive walk-through for SHA and lowest costs for RCA, and (iii) a ‘worst-case’ scenario employing the lowest costs for SHA and highest costs for RCA.

## 3 Results

### 3.1 ICU practice survey and micro-costing study

Of 200 adult general ICUs in England and Wales participating in the CMP in September 2018, 188 (94.0%) completed the survey. Of 182 ICUs that reported using CKRT, 111 (61.0%) had transitioned to RCA (see Supplementary Material for further detail), with 63 ICUs transitioning during the study period.

### 3.2 Patients

After record linkage, exclusion and censoring of patients admitted during the transition period, we included 69,001 patients who received CKRT (60,416 with SHA and 8585 with RCA) in 181 ICUs. 118 ICUs contributed data to the SHA group only, 6 contributing data to the RCA group only, and 57 contributed data pre- and post-transition (Supplementary Fig. 4).

### 3.3 Baseline characteristics

Patients in SHA and RCA groups were similar, with a median age of 67 years (Table 1). Patients in the RCA group were slightly more likely to be male, with slightly more comorbidity and slightly higher predicted mortality. Measures of physiology, before and after multiple imputation, are summarised in Supplementary Table 4. Unadjusted outcomes are summarised in Table 2.

**Table 1:** Baseline characteristics.

Characteristic	All patients		Patients from ICUs contributing data both before and after transition	
	SHA (*N* = 60,416)	RCA (*N* = 8585)	SHA (*N* = 15,373)	RCA (*N* = 7209)
Sex, n (%)				
Female	24,294 (40.2)	3317 (38.6)	6258 (40.7)	2787 (38.7%)
Male	36,122 (59.8)	5268 (61.4)	9115 (59.3)	4422 (61.3%)
Age, median (IQR)	67 (55–75)	67 (55–75)	67 (56–76)	67 (55–75)
Comorbidities identified from previous hospital admission, n (%)				
Congestive cardiac failure	9820 (16.3)	1466 (17.1)	2332 (15.2)	1196 (16.6)
Peripheral vascular disease	6778 (11.2)	978 (11.4)	1667 (10.8)	788 (10.9)
Cerebrovascular disease	2695 (4.5)	435 (5.1)	703 (4.6)	367 (5.1)
Chronic pulmonary disease	11,979 (19.8)	1859 (21.7)	2972 (19.3)	1544 (21.4)
Chronic liver disease	7316 (12.1)	1031 (12.0)	1752 (11.4)	904 (12.5)
Malignancy	9246 (15.3)	1327 (15.5)	2506 (16.3)	1111 (15.4)
Medical history recorded at admission to ICU and present within past 6 months, n (%)				
Haematological malignancy	2552 (4.2)	420 (4.9)	667 (4.3)	376 (5.2%)
Severe immunocompromise	4643 (7.7)	813 (9.5)	1439 (9.4)	720 (10.0%)
Metastatic disease	1414 (2.3)	252 (2.9)	515 (3.4)	218 (3.0%)
Severe liver disease	2591 (4.3)	472 (5.5)	617 (4.0)	420 (5.8%)
Severe respiratory disease or home ventilation	1119 (1.9)	184 (2.1)	331 (2.2)	165 (2.3%)
Very severe cardiovascular disease	1196 (2.0)	219 (2.6)	347 (2.3)	190 (2.6)
Cardiopulmonary resuscitation in previous 24 h, n (%)				
In the community	1503 (2.5)	296 (3.4)	339 (2.2)	251 (3.5)
In hospital	3326 (5.5)	520 (6.1)	876 (5.7)	438 (6.1)
Year of admission to ICU, n (%)				
2009	4668 (7.7)	0 (0.0)	1632 (10.6)	0 (0.0%)
2010	7014 (11.6)	46 (0.5)	2557 (16.6)	0 (0.0%)
2011	7802 (12.9)	73 (0.9)	2877 (18.7)	0 (0.0%)
2012	8042 (13.3)	188 (2.2)	2754 (17.9)	113 (1.6%)
2013	8100 (13.4)	650 (7.6)	2376 (15.5)	567 (7.9%)
2014	7927 (13.1)	1393 (16.2)	1663 (10.8)	1255 (17.4%)
2015	7890 (13.1)	2333 (27.2)	1168 (7.6)	1929 (26.8%)
2016	7365 (12.2)	3018 (35.2)	346 (2.3)	2572 (35.7%)
2017	1608 (2.7)	884 (10.3)	0 (0.0)	773 (10.7%)
Type of ICU admission, n (%) a				
Medical	49,512 (82.0)	7155 (83.4)	12,564 (81.7)	5983 (83.0%)
Elective surgical	2218 (3.7)	261 (3.0)	599 (3.9)	217 (3.0%)
Emergency surgical	8683 (14.4)	1168 (13.6)	2210 (14.4)	1008 (14.0%)
Primary reason for admission to ICU (body system), n (%)				
Cardiovascular	11,549 (19.1)	1953 (22.8)	2959 (19.2)	1588 (22.0)
Dermatological	986 (1.6)	133 (1.6)	210 (1.4)	119 (1.7)
Endocrine, Metabolic, Thermoregulation and Poisoning	3187 (5.3)	435 (5.1)	699 (4.5)	376 (5.2)
Gastrointestinal	11,824 (19.6)	1569 (18.3)	3124 (20.3)	1366 (18.9)
Genito-urinary	15,777 (26.1)	1974 (23.0)	4059 (26.4)	1653 (22.9)
Haematological	1547 (2.6)	230 (2.7)	415 (2.7)	185 (2.6)
Musculoskeletal	1199 (2.0)	180 (2.1)	316 (2.1)	154 (2.1)
Neurological	1739 (2.9)	273 (3.2)	436 (2.8)	249 (3.5)
Respiratory	12,608 (20.9)	1838 (21.4)	3155 (20.5)	1519 (21.1)
First 24 h in ICU				
Mechanically ventilated, n (%)	36,174 (60.0)	5117 (59.7)	9437 (61.5)	4276 (59.4%)
Sepsis−3 criteria met a, n (%)	28,743 (47.6)	4029 (46.9)	7511 (48.9)	3426 (47.5%)
APACHE II acute physiology score, mean (SD) a	18.3 (6.2)	18.2 (6.1)	18.1 (6.2)	18.3 (6.2)
ICNARC model physiology score, mean (SD) a	28.1 (8.9)	28.3 (9.3)	28.0 (8.9)	28.4 (9.3)
ICNARC model predicted mortality probability, mean (SD) a	0.568 (0.248)	0.577 (0.253)	0.572 (0.247)	0.580 (0.252)

APACHE II: Acute Physiology and Chronic Health Evaluation II; ICNARC: Intensive Care National Audit and Research Centre; RCA: regional citrate anticoagulation; SD: standard deviation; SHA: systemic heparin anticoagulation.

a Variable contains some missing data that have been excluded from summary statistics. See Supplementary Table 4 for detailed physiology and missing data.

**Table 2:** Unadjusted outcomes.

Outcome	All patients		Patients from ICUs contributing data both before and after transition		
	SHA (N = 60,416)	RCA (N = 8585)	SHA (N = 15,373)	RCA (N = 7209)	Unadjusted difference (95% CI)
Primary outcome:					
90-day mortality, n (%)	32,174 (53.3)	4634 (54.0)	8289 (53.9)	3977 (55.2)	1.2 (−0.1–2.6)
Secondary outcomes:					
Duration of organ support, calendar days					
Kidney support, mean (SD)	5.3 (6.4)	5.9 (7.2)	5.2 (6.0)	5.9 (6.9)	0.6 (0.5–0.8)
Median (IQR)	3 (2–6)	4 (2–7)	3 (2–6)	4 (2–7)	
Advanced cardiovascular support, mean (SD)	2.5 (3.8)	2.2 (3.5)	2.3 (3.3)	2.0 (3.3)	−0.2 (−0.3−−0.1)
Median (IQR)	1 (0–4)	1 (0–3)	1 (0–3)	1 (0–3)	
Advanced respiratory support, mean (SD)	7.9 (13.1)	7.7 (14.9)	7.8 (12.6)	7.4 (15.1)	−0.3 (−0.7–0.0)
Median (IQR)	3 (0−10)	3 (0–10)	3 (0–10)	3 (0–9)	
Lengths of stay, calendar days					
ICU LOS, mean (SD)	12.7 (15.8)	13.4 (18.3)	12.6 (15.3)	13.0 (17.9)	0.4 (−0.1–0.8)
Median (IQR)	8 (4–15)	8 (4–17)	8 (4–16)	8 (4–16)	
Subsequent hospital LOS, mean (SD)	14.9 (28.9)	13.8 (27.3)	15.4 (31.1)	13.8 (27.8)	−1.6 (−2.5−−0.8)
Median (IQR)	4 (0–18)	3 (0–16)	4 (0–19)	2 (0–16)	
Total hospital LOS, mean (SD)	27.4 (35.9)	27.0 (34.8)	28.0 (37.6)	26.6 (34.8)	−1.3 (−2.4−−0.3)
Median (IQR)	16 (6–35)	16 (6–34)	16 (6–35)	16 (5–34)	
Adverse events, n (%)					
Bleeding episodes in ICU	3922 (6.5)	550 (6.4)	974 (6.3)	461 (6.4)	0.1 (−0.6–0.7)
Thromboembolic episodes up to 90 days post-discharge	2943 (4.9)	448 (5.2)	747 (4.9)	369 (5.1)	0.3 (−0.4–0.9)
ESKD treated by KRT at 90 days a, n (%)	1310 (2.2)	187 (2.4)	322 (2.1)	149 (2.3)	0.2 (−0.2–0.7)
ESKD treated by KRT at one year b, n (%)	1466 (2.7)	162 (3.0)	412 (2.7)	131 (2.9)	0.2 (−0.3–0.8)
Mortality, n (%)					
Acute hospital mortality	30,953 (51.5)	4494 (52.6)	8000 (52.2)	3843 (53.5)	1.3 (−0.1–2.7)
30–day mortality	28,304 (46.8)	4108 (47.9)	7260 (47.2)	3527 (48.9)	1.7 (0.3–3.1)
one-year mortality	35,700 (59.1)	5125 (59.7)	9241 (60.1)	4373 (60.7)	0.5 (−0.8–1.9)
Health economic outcomes:					
Costs, £, mean (SD)					
CKRT	1760 (1926)	2656 (3089)	1722 (1821)	2622 (2924)	900 (838–962)
ICU bed days	24,126 (29,457)	25,180 (30,109)	23,978 (28672)	24,384 (28575)	406 (−395–1208)
Non-ICU hospital bed days	7270 (14,445)	6378 (13,373)	9094 (18296)	8586 (17823)	−508 (−1016−−1)
Dialysis, first year	480 (3169)	535 (3362)	467 (3105)	509 (3278)	42 (−46–131)
Dialysis, lifetime	12,683 (100,819)	14,942 (113,137)	11,979 (96,357)	14,463 (112,139)	2484 (−360–5329)
Total, first year	34,389 (37,321)	36,677 (39,771)	35,261 (38,219)	36,102 (38,900)	840 (−235–1916)
Total, lifetime	45,839 (108,860)	49,157 (120,251)	46,773 (104,803)	50,056 (119,803)	3283 (210–6355)
Benefits, mean (SD)					
QALYs, first year	0.199 (0.227)	0.196 (0.226)	0.195 (0.226)	0.191 (0.225)	−0.004 (−0.011–0.002)
QALYs, lifetime	9.065 (11.470)	9.003 (11.532)	8.799 (11.368)	8.776 (11.463)	−0.024 (−0.342–0.295)

CI: confidence interval; CKRT: continuous kidney replacement therapy; ESKD: end-stage kidney disease; ICU: Intensive Care Unit; IQR: interquartile range; LOS: length of stay; QALY: quality-adjusted life year; RCA: regional citrate anticoagulation; KRT: kidney replacement therapy; SD: standard deviation; SHA: systemic heparin anticoagulation.

a Excludes 2497 patients with incomplete follow-up data.

b Excludes 10,060 patients with incomplete follow-up data.

### 3.4 Primary outcome

**Fig. 1:**
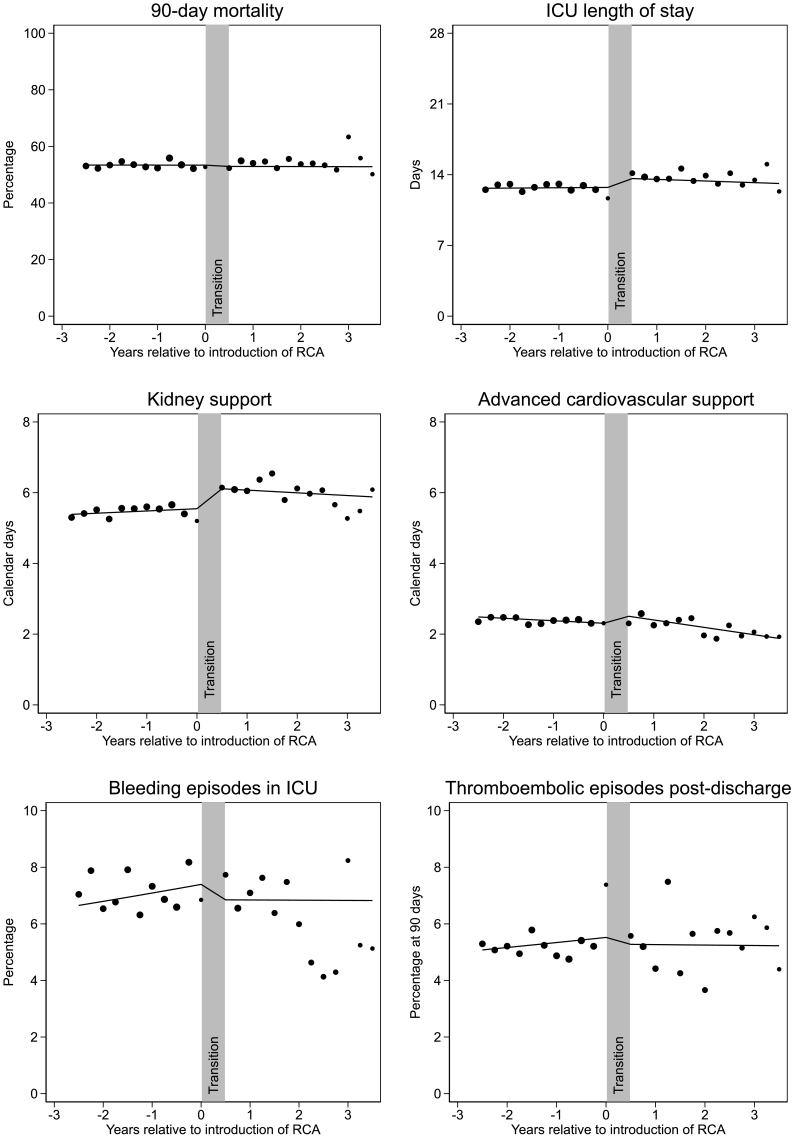
Clinical impacts of transition by intensive care units from systemic heparin anticoagulation (SHA) to regional citrate anticoagulation (RCA) for continuous kidney replacement therapy. Dots represent average values for patients within three-month periods of time. Size of dots represents number of patients. Dots represent only patients from ICUs contributing data both before and after transition (*N* = 22,582) whereas lines represent the marginal estimates of the full regression models including all patients (*N* = 69,001), with adjustment for all covariates, and so cannot be expected to fit the dots exactly.

After adjusting for patient covariates, there were no trends over time pre- or post-transition to RCA, and no step-change in 90-day mortality associated with ICU transition (adjusted OR 0.98, 95% CI 0.89–1.08) (Table 3 and Fig. 1).

**Table 3:** Interrupted time-series analysis (adjusted for all covariates).

Outcome	SHA trend, adjusted OR or difference (Δ) per year (95% CI)	RCA trend, adjusted OR or difference (Δ) per year (95% CI)	Change in trend, adjusted OR or difference (Δ) per year (95% CI)	Step-change, adjusted OR or difference (Δ) (95% CI)	Joint *p* value
Primary outcome:					
90-day mortality, OR	1.00 (0.99–1.01)	1.00 (0.96–1.04)	1.00 (0.96–1.04)	0.98 (0.89–1.08)	0.89
Secondary outcomes:					
Duration of organ support, calendar days, Δ					
Kidney support	0.06 (0.04–0.09)	−0.08 (−0.19–0.04)	−0.14 (−0.26−−0.02)	0.53 (0.28–0.79)	0.00026
Advanced cardiovascular support	−0.07 (−0.08−−0.06)	−0.21 (−0.27−−0.14)	−0.14 (−0.20−−0.07)	0.23 (0.09–0.38)	0.00012
Advanced respiratory support	−0.11 (−0.16−−0.06)	−0.20 (−0.42–0.02)	−0.09 (−0.32–0.14)	0.53 (0.03–1.03)	0.099
Lengths of stay, calendar days, Δ					
ICU LOS	0.03 (−0.03–0.09)	−0.16 (−0.44–0.12)	−0.20 (−0.48–0.09)	0.86 (0.24–1.49)	0.025
Subsequent hospital LOS	−0.29 (−0.40−−0.18)	−0.45 (−0.96–0.05)	−0.17 (−0.68–0.34)	0.41 (−0.71–1.54)	0.75
Total hospital LOS	−0.26 (−0.39−−0.12)	−0.69 (−1.32−−0.07)	−0.44 (−1.08–0.20)	1.26 (−0.14–2.66)	0.20
Adverse events, OR					
Bleeding episodes in ICU	1.05 (1.03–1.06)	1.00 (0.93–1.08)	0.95 (0.88–1.03)	0.90 (0.76–1.06)	0.031
Thromboembolic episodes up to 90 days post-discharge	1.04 (1.02–1.06)	1.00 (0.92–1.08)	0.96 (0.88–1.05)	0.94 (0.78–1.13)	0.21
ESKD treated by KRT at 90 days a	1.02 (0.99–1.05)	1.00 (0.87–1.15)	0.98 (0.85–1.13)	1.04 (0.77–1.41)	0.96
ESKD treated by KRT at one year b	1.05 (1.02–1.08)	0.99 (0.84–1.17)	0.94 (0.79–1.12)	1.00 (0.74–1.36)	0.69
Mortality, OR					
Acute hospital mortality	0.99 (0.98–1.00)	0.99 (0.95–1.03)	1.00 (0.95–1.04)	1.01 (0.92–1.12)	0.97
30–day mortality	1.01 (1.00–1.02)	1.02 (0.98–1.07)	1.01 (0.97–1.06)	0.94 (0.86–1.04)	0.50
One-year mortality	1.01 (1.00–1.01)	0.99 (0.95–1.04)	0.99 (0.95–1.03)	0.96 (0.87–1.06)	0.34
One-year health economic outcomes					
Costs, £*,* Δ	−61 (−198–76)	−696 (−1350−−42)	−635 (−1300−31)	2456 (999–3912)	0.0042
QALYs*,* Δ	−0.000 (−0.001–0.000)	−0.000 (−0.004–0.003)	0.000 (−0.003–0.004)	0.004 (−0.004–0.012)	0.44
INB c, £*,* Δ	53 (−85–191)	692 (34–1350)	639 (−31–1309)	−2376 (−3841−−911)	–

CI: confidence interval; ESKD: end-stage kidney disease; ICU: Intensive Care Unit; IQR: interquartile range; LOS: length of stay; OR: odds ratio; QALY: quality-adjusted life year; RCA: regional citrate anticoagulation; KRT: kidney replacement therapy; SHA: systemic heparin anticoagulation. N = 69,0001.

a Excluding 2497 patients with incomplete follow-up.

b Excluding 10,060 patients with incomplete follow-up.

c INB calculated by multiplying the incremental QALY by £20,000 and subtracting the incremental costs from this.

### 3.5 Secondary outcomes

After adjusting for patient covariates, transition to RCA was associated with step increases in ICU LOS (adjusted difference 0.86 days, 95% CI 0.24–1.49); and calendar days of kidney support (adjusted difference per year 0.53 days, 95% CI 0.28–0.79) and advanced cardiovascular support (adjusted difference per year 0.23 days, 95% CI 0.09–0.38) (Table 3). Secondary mortality outcomes were consistent with the primary outcome (Table 3).

Rates of significant bleeding during hospital stay were increasing over time pre-transition, but were stable post-transition. After adjusting for patient covariates, transition to RCA was associated with a non-significant step decrease in bleeding episodes (adjusted OR = 0.90, 95% CI 0.76–1.06), with joint significance of the step-change and change in trend. Rates of thromboembolism were unaffected by transition. There were no changes in trend or step-changes for development of ESKD in either the prespecified one-year or post-hoc 90-day analyses (Table 3).

### 3.6 Health economic outcomes

Results of the micro-costing study are summarised in Table 2 and detailed in Supplementary Table 2. RCA was associated with higher treatment-related costs, which reflected higher hourly costs of consumables, multiplied by more time spent on CKRT per calendar day (mean, 12.4 h vs 10.4 h) and longer duration of kidney support (mean, 5.94 vs 5.35 calendar days). Differences between groups in accrual of QALYs were negligible (Table 2 and Table 3).

**Fig. 2:**
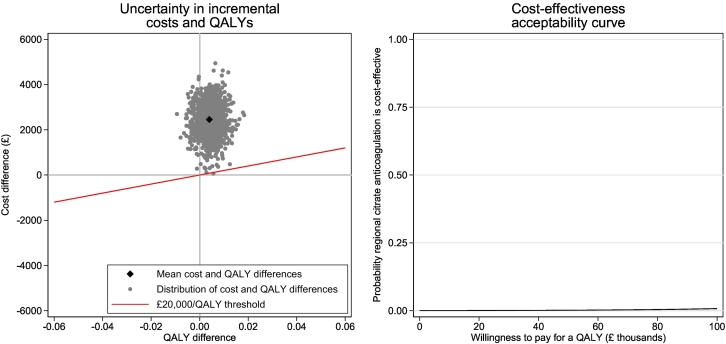
Health economic impacts of transition by intensive care units from systemic heparin anticoagulation (SHA) to regional citrate anticoagulation (RCA) for continuous kidney replacement therapy. Dots represent individual random draws from estimated probability distributions.

After adjusting for patient covariates, transition to RCA was associated with a step-increase in costs to one year of £2456 (95% CI £999–£3912) per patient. Benefits in terms of QALYs at one year were negligible and stable over time. The estimated INB at one year was £ − 2376 (95% CI £ − 3841–£ − 911). The estimated likelihood of cost-effectiveness at one year with a willingness to pay of £20,000/QALY was <0.1% (Fig. 2). This did not increase appreciably at higher thresholds of willingness to pay. Extrapolation to a lifetime horizon indicated a projected step-increase in costs of £4714 (95% CI £496–£8933) with no significant impact on QALYs (Supplementary Table 5).

### 3.7 Subgroup analysis

Analyses of the primary and secondary outcomes for patients meeting Sepsis-3 criteria were consistent with the whole population results (Supplementary Table 6).

### 3.8 Sensitivity analyses

Sensitivity analyses restricted to the 57 ICUs contributing data both before and after the transition to RCA, were consistent with the primary results (Supplementary Table 5). Sensitivity analyses incorporating best- and worst-case treatment costs health differed only in the cost associated with transition (Supplementary Table 5).

## 4 Discussion

In our study of 69,001 patients who received CKRT whilst treated in one of 181 ICUs, transition from SHA to RCA was not associated with a significant step-change or change of trend in 90-day mortality. However, RCA was associated with patients spending more time receiving kidney and cardiovascular support, longer ICU LOS, and was more expensive for hospitals to deliver. Although we found no step change in bleeding episodes, our prespecified joint test, taking in to account an increasing trend with SHA was significant in both our main and sensitivity analyses (though not in the Sepsis-3 cohort).

Our high survey response rate allowed us to include 93% of all general ICUs in England or Wales who could have contributed. We checked transition dates in multiple ways to ensure reliability. Our micro-costing study was based on seven ICUs representing all commonly used CKRT systems. With health-related quality of life data from the multi-center 8000-patient ICON study, our health economic analysis is tailored to the study population. The groups were well matched and we accounted for a wide range of small differences in our adjusted models.

One limitation of our study is that we did not have patient-level data on anticoagulation method received. Although we excluded patients with hepatic failure (the most common reason for use of SHA reported by ICUs using RCA), some patients in the RCA group are likely to have received SHA, other anticoagulants or no anticoagulation. Our results are therefore best interpreted as a pragmatic analysis of what happens to ICU populations when units transition from using SHA to RCA as first-line therapy for the majority of patients, rather than what would happen to individual patients receiving RCA vs SHA. Similarly, we are unable to distinguish between continuous and intermittent techniques for delivering kidney support but both are captured within the definition of kidney support days collected in the CMP.

Although our clinical effectiveness analyses are based on many ICUs, the analysis of CKRT sessions and hours per calendar day was based on data from only two ICUs. Results of our micro-costing study suggest that RCA is more expensive per hour and per session of CKRT; a difference that was then amplified by more time per day and more days spent receiving CKRT. It is important to note that these differences in treatment costs were not drivers of the overall difference in healthcare costs, which principally reflect differences in overall durations of organ support, and in ICU and hospital lengths of stay. As durations of organ support and lengths of stay were observed for the full cohort of 69,001 patients, our findings with respect to overall healthcare costs are likely to be generalisable. The inclusion of broader healthcare costs may explain the contrast between our findings and those of an Australian study that reported little difference in costs relating to consumables and tests only [26].

Since we commenced our work, two further meta-analyses [27,28] and one Cochrane review [29] reported similar findings prior to the 2021 publication of a midsized RCT (*n* = 596) [8]. The RCT was terminated after demonstrating increased filter life span, with limited power to assess differences in the coprimary endpoint of 90-day mortality, for which the trial was deemed futile. Covariate balance in the trial was questionable; patients treated with RCA had lower rates of comorbidity and a 20% higher fluid balance at baseline. Furthermore, while a significant reduction in bleeding was reported (OR 0.27, 95% CI 0.15–0.49), significant increases were observed in risk of metabolic complications (alkalosis and hypophosphataemia), infections (OR 1.71, 95% CI 1.23–2.39) and development of chronic kidney disease at 90 days (OR 2.30, 95% CI 1.09–4.86). It is also notable that, in post hoc analyses, infection was found to be associated with both longer filter life—the same coprimary outcome for which the trial was terminated early for “benefit”—and increased mortality.

In agreement with previous work, we found that RCA resulted in longer filter life; however, we found that this translated into longer, but not fewer, sessions of CKRT. In both our primary and Sepsis-3 cohorts, RCA was associated with increased ICU LOS and kidney and advanced cardiovascular support. In prior studies, bleeding is commonly ill-defined, with ambiguous clinical implications [[30], [31], [32]]. When we took into account an upgoing trend in bleeding episodes in the SHA group in our joint test, we also found decreased bleeding rates with RCA, though we found no significant step change when moving between therapies. Although we would be cautious of over-interpreting our result alone, taken with prior work we think it is likely that an effect on bleeding events exists. However, when placed in the context of no mortality effect, increased time receiving kidney and cardiovascular support and longer ICU LOS, it seems unlikely that this finding translates into significant patient benefit.

Finally, our detailed health economic analysis indicates that transition to RCA results in substantially increased costs over the first year with no evidence of other health economic benefits. In 2019, there were 17,905 patients meeting primary inclusion criteria and receiving CKRT in the CMP; if all ICUs had just transitioned from SHA to RCA, this would have equated to an estimated additional 4297–26,678 ICU bed-days and cost £18 m to £70 m.

## 5 Conclusions

From these prior studies and opinion pieces [[33], [34], [35]], clinicians could believe that because RCA prolongs filter life it is cost-saving, and that fewer bleeding episodes implies clinical benefit. Our population-level study of clinical outcomes and comprehensive analysis of costs indicates that these conclusions may be incorrect. We found no overall evidence that introduction of RCA in England and Wales has improved outcomes for patients, but rather was associated with increased requirements for ICU care and organ support and substantially increased healthcare costs. As such, these findings do not support widespread adoption of RCA. Any future trials comparing RCA and SHA should be designed to accommodate small or nil differences in mortality and to capture the full impacts on healthcare resource use.

## Funding

This work was supported by the UK National Institute for Health and Care Research (grant number 16/111/136).

## Author statements

**JCD**: Data curation, Methodology, Formal analysis, Visualisation, Writing – original draft. **DWG**: Investigation, Project administration, Writing – original draft. **ZS**: Conceptualisation, Methodology, Formal analysis, Funding acquisition, Writing – review & editing. **MB**: Conceptualisation, Data curation, Funding acquisition, Writing – review & editing. **RAH**: Conceptualisation, Funding acquisition, Writing – review & editing. **FJC**: Conceptualisation, Funding acquisition, Writing – review & editing. **LF**: Conceptualisation, Funding acquisition, Writing – review & editing. **RFL**: Conceptualisation, Data curation, Funding acquisition, Writing – review & editing. **CM**: Conceptualisation, Data curation, Funding acquisition, Writing – review & editing. **MO**: Conceptualisation, Data curation, Funding acquisition, Writing – review & editing. **PRM**: Conceptualisation, Data curation, Funding acquisition, Supervision, Writing – review & editing. **DAH**: Conceptualisation, Data curation, Methodology, Formal analysis, Visualisation, Funding acquisition, Supervision, Writing – review & editing. **KMR**: Conceptualisation, Data curation, Funding acquisition, Supervision, Writing – review & editing. **JDY**: Conceptualisation, Data curation, Funding acquisition, Investigation, Writing – original draft. **PJW**: Conceptualisation, Funding acquisition, Writing – original draft.

## Declaration of Competing Interest

MO has received speaker honoraria and research funding from Fresenius Medical, speaker honoraria and research funding from 10.13039/100004702Baxter, and is a member of an advisory board of Fresenius – NxStage. LF has received research funding from 10.13039/100004702Baxter and lecture fees from Baxter and Fresenius. PW was Chief Medical Officer for Sensyne health and holds shares in the company. He declares grants from Wellcome, the National Institute for Health and Care Research, and Sensyne Health during the study period.

## Appendix A Supplementary data

Supplementary material Image 1
