# Supplementary material for: Regional citrate anticoagulation versus systemic heparin anticoagulation for continuous kidney replacement therapy in intensive care
Source: J Crit Care. 2023 Apr;74:None. doi: 10.1016/j.jcrc.2022.154218 (PMC9977605; doi:10.1016/j.jcrc.2022.154218)
Supplement: Supplementary file 1 — Supplementary material [file mmc1.docx]

Regional citrate anticoagulation versus systemic heparin anticoagulation for continuous kidney replacement therapy in intensive care

Supplementary Material

Contents

[ICU practice survey 3](#_Toc120281600)

[Survey methods 3](#_Toc120281601)

[Survey results 3](#_Toc120281602)

[Micro-costing study 5](#_Toc120281603)

[Micro-costing methods 5](#_Toc120281604)

[Analysis of PICRAM data to estimate CKRT system up-time and transfusions 5](#_Toc120281605)

[Cognitive walk-through exercise 5](#_Toc120281606)

[Micro-costing results 5](#_Toc120281607)

[Analysis of PICRAM data to estimate CKRT system up-time and transfusions 5](#_Toc120281608)

[Cognitive walk-through exercise 7](#_Toc120281609)

[Interrupted time-series analysis 9](#_Toc120281610)

[Supplementary methods 9](#_Toc120281611)

[Definitions and codes 9](#_Toc120281612)

[Data cleaning and linkage 11](#_Toc120281613)

[Statistical analysis (additional detail) 11](#_Toc120281614)

[Supplementary tables and figures 12](#_Toc120281615)

[Supplementary Material references 16](#_Toc120281616)

# ICU practice survey

## Survey methods

The aim of the survey was to identify which ICUs had transitioned from systemic heparin anticoagulation (SHA) to regional citrate anticoagulation (RCA) for continuous kidney replacement therapy (CKRT), and when the transition occurred. We used the SurveyMonkey^®^ online platform (www.surveymonkey.com) to ask Clinical Directors/lead clinicians of all adult general ICUs in England and Wales (identified via the Case Mix Programme national clinical audit of adult critical care) whether their ICU was using SHA, RCA or both.

ICUs using SHA were asked their current effluent flow rate and which CKRT system was in use. ICUs using RCA were asked their dates corresponding to start and completion of transition to RCA, their effluent flow rate before and following transition, and which CKRT system in use before and following transition. ICUs using both RCA and SHA were asked the dates corresponding to start and completion of transition, their effluent flow rate before and following the transition, the CKRT system in use for SHA and for RCA, the proportion of patients receiving SHA, and the conditions/indications for which SHA is preferred.

For ICUs completing the switch from CKRT to RCA before 01 January 2017, we confirmed the switch dates by requesting purchase details of the consumables involved in CKRT (including anticoagulants and fluids) and/or requesting the clinician to cross-check on computerised information systems. Where this information conflicted with the original survey entry, we contacted sites for further clarification.

## Survey results

We identified 200 adult general ICUs in England and Wales as participating in the CMP in September 2018. Between September 2018 and March 2019, 188 (94%) ICUs completed the online survey. Of these, 182 ICUs (96.8%) reported using CKRT, of which 111 (61%) reported transitioning from SHA to RCA (Supplementary Figure 1). Of the 182 ICUs that reported using CKRT (Supplementary Figure 1), 181 (99.5%) contributed data for at least one patient to the study cohort. One ICU did not provide a date corresponding to the start of the change to RCA and was therefore excluded. Of the 181 complete responses, 175 (96.7%) ICUs contributed patients for the period that SHA was in use and 63 (34.8%) contributed patients for the period that RCA was in use. 57 ICUs contributed patients for both SHA and RCA periods. Characteristics of the units are presented in Supplementary Table 1.

Eligible adult general ICUs in England and Wales

n=200

Completed online survey
n=188 (94.0%)

Do use CKRT
n=182 (96.8%)

Use SHA for CKRT
n=71 (39.0%)

Use RCA for CKRT
n=111 (61.0%)

Do not use CKRT
n=6 (3.2%)

Contributing data to RCA period only
n = 6

Contributing data to SHA period only
n = 118

Contributing patient data to both SHA and RCA periods n = 57

Missing RCA start date
n=1 (0.9%)

Changed to RCA after study period n=47 (42.3%)

Eligible adult general ICUs in England and Wales

n=200

Completed online survey
n=188 (94.0%)

Do use CKRT
n=182 (96.8%)

Use SHA for CKRT
n=71 (39.0%)

Use RCA for CKRT
n=111 (61.0%)

Do not use CKRT
n=6 (3.2%)

Contributing data to RCA period only
n = 6

Contributing data to SHA period only
n = 118

Contributing patient data to both SHA and RCA periods n = 57

Missing RCA start date
n=1 (0.9%)

Changed to RCA after study period n=47 (42.3%)

Supplementary Figure 1 Flow chart of the response to survey of anticoagulation practice

**Supplementary Table 1 Summary of responses for ICUs according to the mode of anticoagulation reported**

|  | **Anticoagulation mode** | |
| --- | --- | --- |
|  | **SHA (n = 71)** | **RCA (n = 111)** |
| Number of beds, median (IQR) | 12.0 (9.0–16.5) | 14.0 (10.0–18.0) |
| Number of CKRT machines, median (IQR) | 4.0 (3.0,5.5) | 4 (3–6) |
| Machines per bed ratio, median (IQR) | 0.3 (0.25–0.4) | 0.3 (0.25–0.4) |
| Units reporting, n (%) |  |  |
| 0–5 machines | 53/71 (74.6) | 79/110 (71.8) |
| 6–10 machines | 14/71 (19.7) | 27/110 (24.5) |
| 11–15 machines | 1/71 (1.4) | 2/110 (1.8) |
| 16–20 machines | 1/71 (1.4) | 2/110 (1.8) |
| >20 machines | 2/71 (2.8) | 0/110 (0.0) |
| CKRT system in use, n (%) |  |  |
| Prismaflex (Baxter) | 46/70 (65.7) | 66/110 (60.0) |
| Multifiltrate (Fresenius) | 6/70 (8.6) | 32/110 (29.1) |
| Aquarius (Nikkiso) | 15/70 (21.4) | 11/110 (10.0) |
| Other | 3/70 (4.3) | 1/110 (0.9) |
| Most frequent effluent flow rate in use, n (%) |  |  |
| <20 ml/kg/hour | 2/70 (2.9) | 2/107 (1.8) |
| 25 ml/kg/hour | 14/70 (20.0) | 43/107 (40.2) |
| 30 ml/kg/hour | 12/70 (17.1) | 22/107 (20.6) |
| 35 ml/kg/hour | 31/70 (44.3) | 29/107 (27.1) |
| 40 ml/kg/hour | 0/70 (0.0) | 3/107 (2.8) |
| 45 ml/kg/hour | 0/70 (0.0) | 1/107 (0.9) |
| >45 ml/kg/hour | 0/70 (0.0) | 0 (0.0) |
| Other | 11/70 (15.7) | 7/107 (6.5) |

RCA: regional citrate anticoagulation; SHA: systemic heparin anticoagulation.

# Micro-costing study

## Micro-costing methods

The micro-costing study involved (i) analysis of data from the Post Intensive Care Risk-Adjusted Alerting and Monitoring (PICRAM) study of all patients treated on both Oxford general ICUs from 2009–2015, to estimate the frequency of CKRT system failures and the average number of transfusion episodes for patients receiving SHA or RCA, and (ii) a cognitive walk-through exercise with clinicians from eight ICUs representing the different kidney replacement systems in common use to estimate the staff time and consumables used in SHA or RC. These were combined with staff time costs from the Unit Costs of Health and Social Care [1], unit costs of anticoagulation drugs from the NHS Business Services Authority Drug Tariff [2], and CKRT fluid costs from manufacturers’ quoted prices. NHS Supply Chain costs were used for consumables [3].

### Analysis of PICRAM data to estimate CKRT system up-time and transfusions

CKRT system set-up time and the frequency of system failures were accounted for estimated from anonymised patient data from the Post Intensive Care Risk-Adjusted Alerting and Monitoring (PICRAM) study (containing anonymised electronically-held records of all patients treated on both Oxford general ICUs from 2009–2015) [4, 5] and from the electronic clinical information system for patients treated in Oxford following completion of PICRAM (2015–2017).

We applied the same selection criteria as were used to select records from the CMP database for the clinical effectiveness analysis. We derived the mean number of transfusion episodes, and mean number of transfusion hours, per calendar day of CKRT for each patient from the recorded start and stop times for each transfusion episode. We then used linear regression models to estimate the means of means for patients receiving SHA and patients receiving RCA, controlling for background trends in calendar time.

### Cognitive walk-through exercise

The cognitive walk-through exercise was completed by clinicians from eight ICUs (four using SHA and four using RCA). Clinicians were asked to complete an hierarchical task analysis of CKRT, including conducting initial tests, placing vascular access, preparing, connecting, running and disconnecting the CKRT machine, and finally removing vascular access [6]. We categorised cost items as either staff time or consumables; and as accumulating per patient, per session of CKRT, or per hour of CKRT. Consumables included vascular access catheters, local anaesthetics, gloves, saline ampuoles, saline bags, cleaning products, giving sets/lines, CKRT circuits (including filters), line insertion packs, heparin, syringes, sharps, replacement fluids, effluent bags, calcium solutions, dressings, laboratory tests done prior to insertion or removal of access catheters, connectors and caps, and other miscellaneous items. We then aggregated costs within ICUs and averaged the aggregated costs across ICUs. The highest and lowest aggregate values were also retained for use in sensitivity analyses.

## Micro-costing results

### Analysis of PICRAM data to estimate CKRT system up-time and transfusions

Using the PICRAM database, we identified 692 patients treated with 3241 sessions of SHA-CKRT between January 2009 and June 2016, and 199 patients treated with 911 sessions of RCA-CKRT between October 2016 and August 2019.

Models evaluated at the date of commencement of RCA (after censoring of a transition period) indicated a small, non-significant difference in CKRT sessions per calendar day of (SHA = 0.705, RCA = 0.736, difference = 0.031, 95% CI −0.005–0.066; Supplementary Figure 2). We therefore adopted an equal average expectation of 0.721 in the cost-effectiveness analysis.

In contrast, we estimated an increase from an average of 10.4 hours of CKRT per calendar for SHA to 12.4 hours of CKRT per calendar day for RCA (difference = 2.1, 95% CI 1.7–2.4). We considered this difference both clinically and statistically significant and so incorporated separate estimates into the cost-effectiveness analysis.


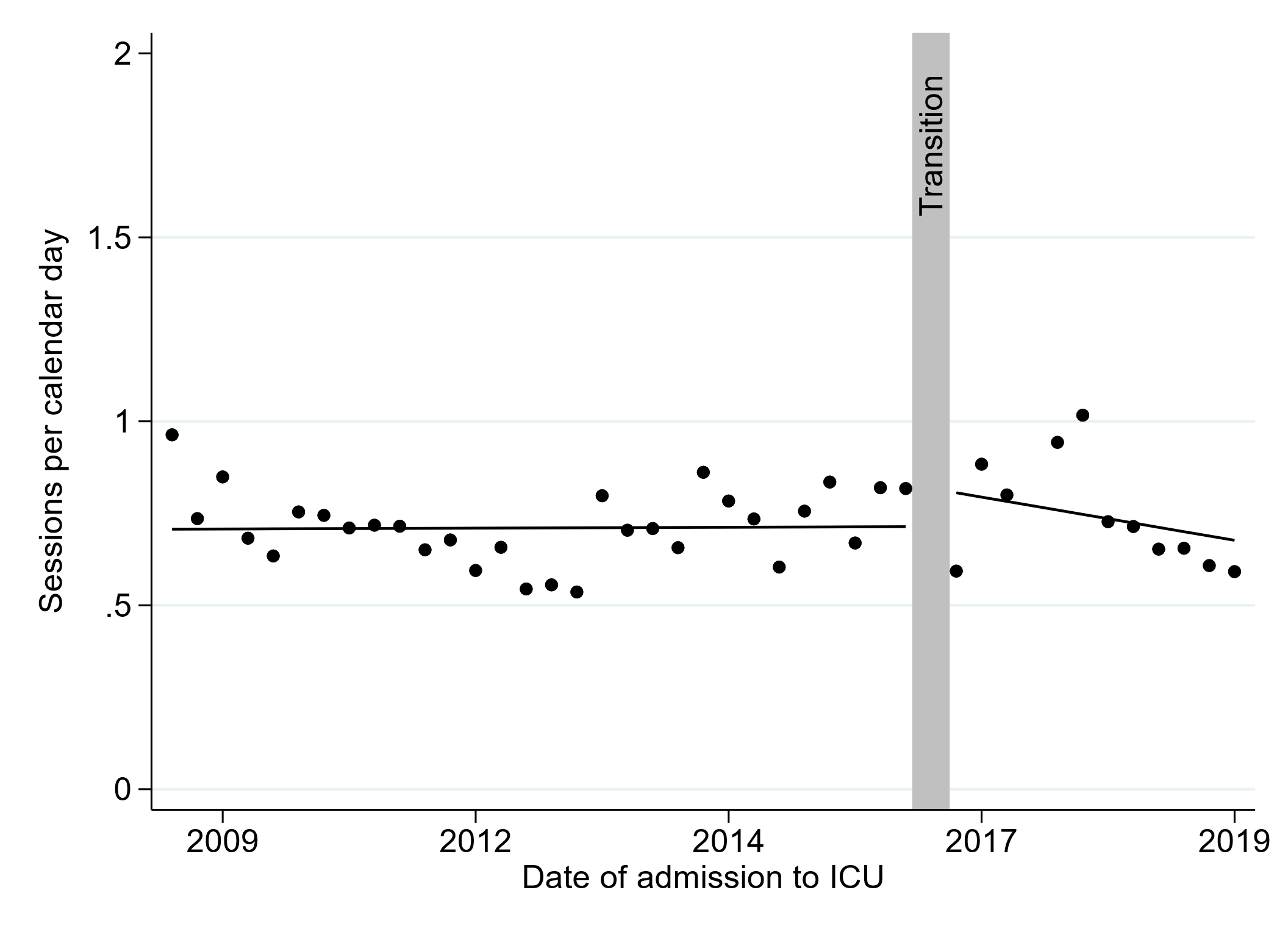


Supplementary Figure 2 Mean number of CKRT sessions per calendar day^*^ of CKRT, by method of anticoagulation

* The number of CKRT sessions per calendar day, and hours per calendar day, are related of filter life but include time not on CKRT accrued during days of CKRT commencement and cessation. These measures were constructed specifically their compatibility with duration of kidney support, which is measured in calendar days, and should not be interpreted as measures of filter life.


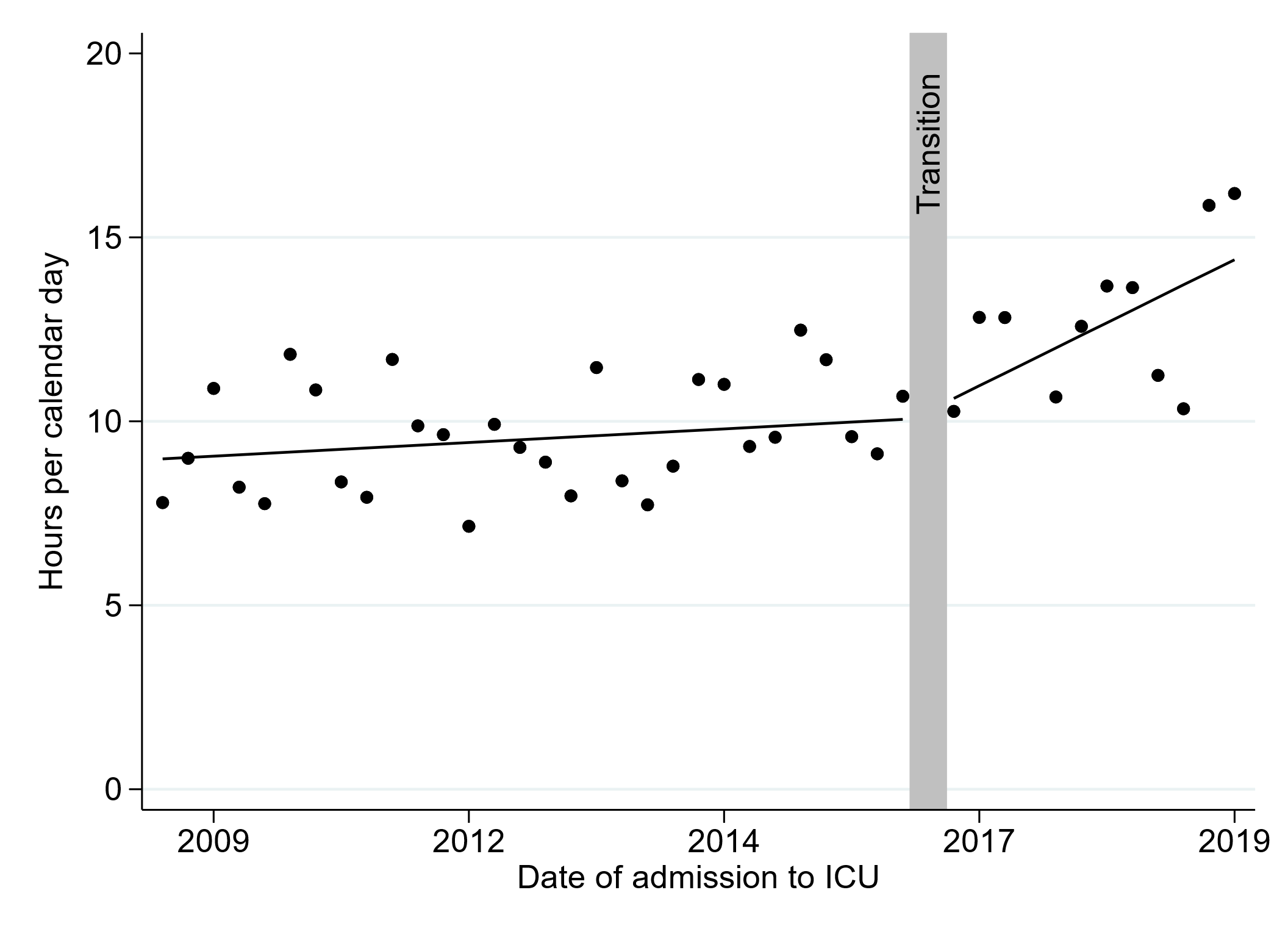


Supplementary Figure 3 Mean number of hours of CKRT per calendar day* of CKRT, by method of anticoagulation

* The number of CKRT sessions per calendar day, and hours per calendar day, are related of filter life but include time not on CKRT accrued during days of CKRT commencement and cessation. These measures were constructed specifically their compatibility with duration of kidney support, which is measured in calendar days, and should not be interpreted as measures of filter life.

### Cognitive walk-through exercise

Staff from seven ICUs participated in the cognitive walk-through exercise between April and July of 2019. Three of the ICUs used SHA (two of which used Prismaflex (Baxter) machines and one used Multifiltrate (Fresenius) machine) and four used predominantly RCA (two of which used Prismaflex (Baxter) machines and two of which used Multifiltrate (Fresenius) machines).

Results from the cognitive walk-through are summarised in Supplementary Table 2. For illustration purposes, these have been combined with results from the analysis of PICRAM and CMP data to produce expected totals per patient (please note that the interrupted time-series analysis incorporated patient-level data on calendar days of CKRT provided). Estimated costs were more variable in the sites using SHA but comparison of the means, lowest and highest values between groups painted a consistent picture of slightly lower costs related to preparing the patient for RCA but substantially higher costs in terms preparing and running the machine. Holding the amount of CKRT (calendar days and hours per day) constant, we estimated RCA to cost an additional £509 per patient (or between £97 less and £1073 more, comparing minima with maxima). Factoring in the observed differences in CKRT up-time from analysis of PICRAM (hours of CKRT per calendar day of CKRT) and CMP (calendar days of CKRT per patient), we estimated an expected additional cost of £897 per patient (or between £310 and £1432 comparing best and worst cases). The main driver of the overall difference in costs was the difference in cost of consumables required to prepare and run the machine.

Supplementary Table 2 Results of the micro-costing study

|  | **Mean estimated cost, 2019£ (range)** | | | |
| --- | --- | --- | --- | --- |
|  | **SHA** | | **RCA** | |
| Cost component |  |  |  |  |
| Per admission |  |  |  |  |
| Consumables ^a^ |  |  |  |  |
| Initial Tests | 0.64 | (0.00–2.57) | 0.19 | (0.00–0.57) |
| Placing vascular access | 103.61 | (86.54–143.00) | 80.38 | (68.41–87.56) |
| Removing vascular access | 5.39 | (1.98–9.09) | 4.04 | (1.94–8.12) |
| Staff |  |  |  |  |
| Initial Tests | 1.12 | (0.00–4.50) | 1.50 | (0.00–4.50) |
| Placing vascular access | 26.19 | (24.39–28.89) | 32.25 | (25.36–41.64) |
| Removing vascular access | 6.97 | (4.50–8.99) | 5.99 | (4.50–8.99) |
| Subtotal | 143.92 | (123.95–184.20) | 124.35 | (121.36–126.17) |
| Per session of CKRT |  |  |  |  |
| Consumables ^a^ |  |  |  |  |
| Preparing the machine | 176.37 | (111.93–263.17) | 246.44 | (226.71–274.99) |
| Connecting | 2.38 | (1.51–3.76) | 5.68 | (2.32–7.72) |
| Running (fixed) | 2.22 | (0.00–5.14) | 2.57 | (0.00–5.14) |
| Disconnecting | 5.33 | (0.00–9.64) | 4.43 | (3.73–4.96) |
| Staff |  |  |  |  |
| Preparing the machine | 23.85 | (20.23–26.97) | 18.21 | (11.24–27.87) |
| Connecting | 4.16 | (2.25–5.42) | 5.11 | (4.50–5.42) |
| Running (fixed) | 2.25 | (0.00–4.50) | 3.00 | (0.00–4.50) |
| Disconnecting | 11.28 | (6.74–18.15) | 11.99 | (4.50–17.98) |
| Subtotal | 227.85 | (157.99–316.70) | 297.42 | (259.26–330.84) |
| Per hour |  |  |  |  |
| Consumables ^a^ | 8.85 | (7.75–10.85) | 13.23 | (10.52–14.63) |
| Staff | 4.44 | (2.25–6.54) | 3.90 | (2.70–4.50) |
| Subtotal | 13.30 | (10.00–15.34) | 17.13 | (15.02–19.04) |
| Expected total per patient, holding durations of CKRT constant ^b^ | | | | |
| Consumables ^a^ | 1435.94 | (1103.70–1913.60) | 1988.30 | (1767.18–2195.63) |
| Staff | 488.95 | (402.88–595.25) | 445.93 | (384.12–552.52) |
| Expected grand total | 1924.88 | (1506.58–2416.31) | 2434.22 | (2319.70–2579.75) |
| Expected total per patient, including observed differences in durations of CKRT^2^ | | | | |
| Consumables ^a^ | 1318.80 | (1008.87–1760.29) | 2167.08 | (1918.95–2393.49) |
| Staff | 441.26 | (371.49–530.71) | 490.44 | (418.56–605.39) |
| Expected grand total | 1760.06 | (1380.36–2214.63) | 2657.52 | (2524.35–2812.05) |

^a^ Consumables included vascular access catheters, local anaesthetics, gloves, saline ampuoles, saline bags, cleaning products, giving sets/lines, CKRT circuits (including filters), line insertion packs, heparin, syringes, sharps, replacement fluids, effluent bags, calcium solutions, dressings, laboratory tests done prior to insertion or removal of access catheters, connectors and caps, and other miscellaneous items. ^b^ Expected totals are provided for illustration only; the cost-effectiveness analysis incorporated patient-level observations of calendar days of CKRT. To produce these expected totals, component costs were multiplied by 0.721 CKRT sessions per calendar day of CKRT (both SHA and RCA), 10.4 hours per calendar day (SHA) or 12.4 hours per calendar day (RCA), and 5.35 calendar days per admission (SHA) or 5.94 calendar days per admission (RCA); or by means of these when holding durations of CKRT constant between treatment groups.

# Interrupted time-series analysis

## Supplementary methods

Supplementary Table 3 Description of datasets for linkage and key variables

|  | **Description** | **Key variables** |
| --- | --- | --- |
| Case Mix Programme (CMP) | National clinical audit of patient outcomes from adult, general critical care units (intensive care and combined intensive care/high dependency units) in England, Wales and Northern Ireland which contains validated pooled case mix and outcome data from over 2 million critical care admissions. Since 2015, the CMP has had 100% coverage of adult, general ICUs. | Patient level information including patient identifiers required only for linkage; dates and methods of admission to hospital and ICU; patient case mix (including illness severity scores and mortality probability, derived using standard models for clinical audit); patient outcome at discharge from ICU and hospital; and ICU activity including the number, duration, and level (basic/advanced) of organs supported and length of stay in the ICU and hospital. |
| Civil Registrations (Mortality) dataset [previously Office for National Statistics] | Mortality information, for all deaths registered in England and Wales. | Data including date of death; cause of death; and place of death. |
| Hospital Episodes Statistics for England (HES) | Admission, emergency department attendances and outpatient appointment data at NHS hospitals in England . | Patient level information including patient information (age, gender and ethnicity); diagnoses and operations; dates and methods of hospital admission and discharge; and geographical information (where patients were admitted/treated and live) (1). |
| Patient Episodes Data for Wales (PEDW) | Admission and day case activity in NHS Wales hospitals data and for Welsh residents treated in English Trusts. | Patient level information including patient information (age, gender and ethnicity); diagnoses and operations; and dates and methods of hospital admission and discharge. |
| UK Renal Registry (UKRR) | Data on all patients on KRT including patients receiving haemodialysis, peritoneal dialysis, kidney transplant for ESKD. | Treatment data including date first seen by a kidney physician; date of first ever KRT and modality (for ESKD patients); data on treatment timelines and haemodialysis sessions; and transplant information. |

### Definitions and codes

#### Exclusion criteria: Pre-existing End-stage kidney disease (ESKD), prior transplant or nephrectomy

Pre-existing ESKD, prior transplant or nephrectomy were identified by having a linked record in the UKRR with a date of first ever KRT for ESKD or date of transplant prior to the date of admission to ICU; and/or presence in HES/PEDW indicating ESKD, kidney transplant or nephrectomy, prior to the date of admission to ICU (ICD-10 codes: I12.0, I12.9, N18.6, Z94.0, Z99.2; OPCS codes: M02.2, M02.3, M02.4, M02.5, M02.6, M02.7, M02.8, M02.9, M03.8, M03.9).

#### Outcomes:

Mortality was derived from the difference between date of admission to ICU recorded in the Case Mix Programme and date of death recorded in either the Case Mix Programme or linked death registration data.

ICU and hospital lengths of stay were obtained from dates of admission and discharge recorded in the Case Mix Programme. Durations of organ support are recorded in calendar days in the Case Mix Programme. Kidney support days are any days on which the patient received any acute kidney replacement therapy.

Bleeding episodes during the ICU stay were identified from linked HES/PEDW records (using ICD-10 codes R04, I61, I62, K25.0, K25.2, K25.4, K25.6, K26.0, K26.2, K26.4, K26.6, K27.0, K27.2, K27.4, K27.6, K28.0, K28.2, K28.4, K28.6, K29.0, K92.0, K92.1, K92.2).

Thromboembolic episodes occurring within 90 days after ultimate hospital discharge were identified using identified from linked HES/PEDW records (ICD-10 codes: I26.9, I26.0, I60, I61.0, I61.1, I61.2, I61.3, I61.4, I61.5, I61.6, I61.8, I61.9, I62.0, I62.1, I62.9, I80).

#### Covariates

The covariates included were:

- Age (linear), from CMP
- Comorbidities identified from HES/PEDW based on the Royal College of Surgeons adaptation of the Charlson comorbidity index:
  - Congestive cardiac failure (ICD-10 codes: I11, I13, I42, I43, I50, I25.5, I51.7)
  - Peripheral vascular disease (ICD-10 codes: I70, I71, I72, I73, I77.0, I77.1, K55.1, K55.8, K55.9, R02, Z95.8, Z95.9)
  - Cerebrovascular disease (ICD-10 codes: G45, G46, I6)
  - Chronic pulmonary disease (ICD-10 codes: I26, I27, J40, J41, J42, J43, J44, J45, J46, J47, J60, J61, J62, J63, J64, J65, J66, J67, J68.4, J70.1, J70.3)
  - Chronic liver disease (ICD-10 codes: B18, I85, I86.4, I98.2, K70, K71, K72.1, K72.9, K76, R16.2, Z94.4)
  - Malignancy (ICD-10 codes: (ICD-10 codes: C00–C09, C10–C26, C30–C34, C37–C41, C43, C45–C58, C60–C76, C80–C85, C88, C90–C97)
- Severe conditions in the past medical history, from CMP:
  - Haematological malignancy
  - Severe immunocompromise (radiotherapy, chemotherapy or daily high-dose steroid treatment during the six months prior to ICU admission, or recording of HIV/AIDS or a congenital immunohumoral or combined immune deficiency state)
  - Metastatic disease
  - Severe liver disease (biopsy proven cirrhosis, portal hypertension or hepatic encephalopathy)
  - Severe respiratory disease (shortness of breath with light activity or a requirement for home ventilation)
  - Very severe cardiovascular disease (New York Heart Association functional class IV)
- Dependency prior to admission to acute hospital (no, some or total requirement for assistance with activities of daily living), from CMP
- Body mass index (restricted cubic splines with 3 knots), from CMP
- Location prior to admission to the ICU (emergency department or not in hospital, other hospital (not critical care), other critical care unit, planned admission from theatre following elective/scheduled surgery, unplanned admission from theatre following elective/scheduled surgery, admission from theatre following urgent/emergency surgery, ward or intermediate care area), from CMP
- Cardiopulmonary resuscitation (in hospital or out of hospital) within 24 hours prior to ICU admission, from CMP
- Primary reason for admission to ICU (body system), from CMP
- Receipt of mechanical ventilation during the first 24 hours in ICU, from CMP
- Physiology recorded during the first 24 hours in the ICU, from CMP:
  - Highest heart rate (restricted cubic splines with 4 knots)
  - Lowest systolic blood pressure (restricted cubic splines with 4 knots)
  - Highest central temperature, or highest non-central temperature +1°C if no central temperature recorded (restricted cubic splines with 4 knots)
  - Lowest respiratory rate (right-restricted cubic splines with 4 knots)
  - Total urine output (restricted cubic splines with 4 knots)
  - Lowest total Glasgow Coma Scale (GCS) score (3, 4–6, 7–13, 14, 15, or sedated for entire of first 24 hours)
  - PaO_2_/FiO_2_ from the arterial blood gas with the lowest PaO2 (restricted cubic splines with 4 knots)
  - Lowest pH (restricted cubic splines with 4 knots)
  - PaCO_2_ from the arterial blood gas with the lowest pH (restricted cubic splines with 3 knots)
  - Highest blood lactate (restricted cubic splines with 4 knots)
  - Highest creatinine (right-restricted cubic splines with 4 knots)
  - Highest urea (restricted cubic splines with 4 knots)
  - Highest sodium (restricted cubic splines with 3 knots)
  - Lowest white blood cell count (restricted cubic splines with 4 knots)
  - Lowest platelet count (restricted cubic splines with 4 knots)

#### Healthcare costs

Unit costs of ICU/hospital length of stay and dialysis were obtained from the NHS Reference Costs 2017–18 [7] calculated as a total costs per patient to one year following the index ICU admission. The cost of KRT for ESKD was also estimated for patients identified from UKRR as having ESKD treated using KRT, based on an assumption of three dialysis sessions per week using unit costs of dialysis from the NHS Reference Costs 2017–18.

#### Health-related quality of life and quality adjusted life years

EuroQuol EQ-5D-3L health-related quality of life (HRQoL) data for patients at three months and one year after ICU discharge was obtained from the 8000 patient UK Intensive Care Outcome Network Study (ICON) study [8]. Eligible patients meeting the inclusion criteria were identified and divided into quartiles of age. Averaged EQ-5D-based utility weights were calculated by age quartile at three months and one year and used as the measure of HRQoL. All patients developing ESKD and requiring dialysis were assigned a decrement of 0.14–their HRQoL based on European norms [9] compared against the equivalent age group from the Health Survey for England 2016. This decrement was applied from the date of first KRT for ESKD forward. HRQoL at three months and one year were then combined with the survival data to calculate quality-adjusted life years (QALYs) at one year, assuming zero HRQoL at baseline and death, and linear interpolation between the timepoints.

### Data cleaning and linkage

NHS Digital (https://digital.nhs.uk), acting as a ‘trusted third party’, linked individual patient data from the CMP with data held by the UK Renal Registry (UKRR), Hospital Episode Statistics (HES) and Civil Registrations (deaths) datasets. We uploaded patient identifiers (with no associated clinical data) from the CMP and UKRR clinical audits to secure servers at NHS Digital. In turn, NHS Digital used NHS numbers to match records between the CMP, UKRR, HES and Civil Registrations datasets and return to each data provider their local identifier (a field that is unique to the records within that dataset) together with a common key used to link all records of the same patient across datasets. Following linkage of the patient records, the UKRR and NHS Digital transferred the agreed pseudonymised data to the ICNARC CTU (including the common key) for successfully linked patients. Records with no link to HES/PEDW, or with discordant dates of death, were excluded from analysis.

### Statistical analysis (additional detail)

The multiple imputation model included all covariates planned to be included in the substantive model, with the same functional form, plus the intervention and outcome measures. To ensure reproducibility of results, the random number seed was set prior to producing the imputed datasets.

For the health economic analysis, separate multilevel models were fitted for costs and QALYs, adjusted for the same patient covariates as the models for clinical effectiveness. Incremental net benefit (INB) at one year associated with a change from SHA to RCA was estimated valuing incremental QALYs according to a NICE recommended threshold willingness-to-pay for a QALY gain (£20,000) and subtracting from this the incremental costs. The INB calculation assumed no correlation between costs and QALYs; a single-level, bivariate seemingly-unrelated-regression model for costs and QALYs indicated that the impact of not accounting for correlation would be small and conservative (~3% increase in standard error of INB).

We projected lifetime cost-effectiveness by summarising the relative effects of the alternative strategies on long-term survival and HRQoL, as compared with that of age-gender matched general population [10, 11]. There is evidence to suggest that critical care survivors face a higher probability of death, and lower quality of life after the critical care episode than the general population [11-13]. However, there is no clear evidence as to the duration of this excess mortality; the strongest evidence is in support of an excess mortality of up to five years. The long-term survival of each patient in the RRAM study was calculated from observed survival within one year and their predicted survival from age-gender matched general population survival by applying excess death rates for year 2–5, after which we assumed that all cause death rates were those of the age-gender matched general population. HRQoL of RRAM patients at one year was approximately 73% of that of the age-gender matched general population. We assumed that HRQoL would improve over 5 years to match age-gender matched HRQoL. After five years we applied HRQoL values for the age-gender matched general population [14]. Lifetime QALYs were reported by combining life years and HRQoL. Lifetime costs were projected by applying chronic morbidity costs associated with use of dialysis for ESKD over the lifetime. No further differential costs between the groups after one year were considered.

## Supplementary tables and figures

Hospital Episode Statistics for England (HES)

1,852,825 records with links to
89,463 ICU admissions for
83,805 patients

Office for National Statistics Mortality (ONS)

102,967 records with links to
67,844 ICU admissions for
63,798 patients

United Kingdom Renal Registry (UKRR)

6289 records with links to
7391 ICU admissions for
6242 patients

Patient Episode Database for Wales (PEDW)

62,182 records with links to

7087 ICU admissions for
6651 patients

Case Mix Programme (CMP)

99,945 ICU admissions
met primary inclusion criteria

Secondary exclusions

23,395 (23.4%) ICU admissions for
excluded for overlapping reasons:

- 2783 (2.8%) ICU admissions
  with missing NHS numbers
- 1901 (1.9%) ICU admissions for
  1792 (2.0%) patients with NHS number but no linked health records
- 8038 (8.0%) ICU admissions for
  7504 (8.2%) patients whose hospital did not participate in survey
- 447 (0.5%) ICU admissions for
  418 (0.5%) patients whose hospital returned incomplete survey responses
- 6 (<0.1%) ICU admissions for
  2 (<0.1%) patients with discrepant information about date of death
- 11,484 (11.5%) ICU admissions for
  9932 (10.9%) patients with evidence of prior kidney replacement therapy, nephrectomy or transplant in linked data

Initially treated with RCA-CKRT

8585 (12.4% of uncensored) patients

Initially treated with SHA-CKRT

60,416 (87.6% of uncensored) patients

Treatment-censored records

3400 (4.7%) patients whose index admission overlapped with unit's transition period

Included ICU admissions

76,550 ICU admissions for 72,401 patients

Did not cross over

8548 (99.0%) patients only received RCA-CKRT

Did not cross over

60,339 (99.5%) patients only received SHA-CKRT

Potentially crossed over

83 (1.0%) patients, including:

- 65 (0.8%) patients later received SHA-CKRT and/or
- 18 (0.2%) patients later received CKRT during transition period

Potentially crossed over

323 (0.5%) patients, including:

- 171 (0.3%) patients later received SHA-CKRT and/or
- 157 (0.3%) patients later received CKRT during transition period

Uncensored records

69,001 (95.3%) patients with index admission outside of unit's censoring period

Supplementary Figure 4 Data linkage and cohort construction

Supplementary Table 4 Physiology variables and multiple imputation

|  | Missing, n (%) | Full cohort, available case, mean (SD) | Full cohort, imputed, mean (SD) | SHA, imputed, mean (SD) | RCA, imputed, mean (SD) |
| --- | --- | --- | --- | --- | --- |
| Body mass index, kg/m^2^ | 6554 (9.5) | 28 (7.6) | 28 (7.6) | 28 (7.7) | 28 (7.3) |
| Heart rate (highest), bpm | 125 (0.18) | 116 (25) | 116 (25) | 116 (25) | 115 (25) |
| Systolic blood pressure (lowest), mmHg | 125 (0.18) | 88 (20) | 88 (20) | 88 (20) | 87 (21) |
| Body temperature (highest), °C | 171 (0.25) | 38 (1.2) | 38 (1.2) | 38 (1.2) | 37 (1.2) |
| Respiratory rate (lowest), bpm | 144 (0.21) | 14 (5.4) | 14 (5.4) | 14 (5.5) | 14 (4.7) |
| PaO_2_/FiO_2_ ratio | 2287 (3.3) | 198 (110) | 199 (111) | 199 (111) | 202 (112) |
| Urine output, ml/day | 852 (1.2) | 940 (1166) | 939 (1166) | 937 (1168) | 951 (1151) |
| Serum creatinine (highest), μmol/l | 782 (1.1) | 322 (278) | 322 (278) | 323 (278) | 318 (282) |
| Serum urea, mmol/l (highest) | 757 (1.1) | 20 (14) | 20 (14) | 20 (14) | 20 (14) |
| Serum sodium, mmol/l (highest) | 417 (0.6) | 138 (6.3) | 138 (6.3) | 138 (6.3) | 139 (6.4) |
| Arterial blood pH (lowest) | 1767 (2.6) | 7.2 (0.15) | 7.2 (0.15) | 7.2 (0.15) | 7.2 (0.15) |
| Associated PaCO2 from arterial blood gas with lowest pH/H+, mmHg | 2287 (3.3) | 45 (18) | 45 (18) | 45 (18) | 45 (18) |
| Blood lactate (highest), mmol/l | 2307 (3.3) | 5.2 (4.7) | 5.1 (4.7) | 5.1 (4.7) | 5.2 (4.9) |
| White blood cell count (lowest), ×10^9^/l | 759 (1.1) | 13 (12) | 13 (12) | 13 (13) | 13 (12) |
| Platelet count (lowest) , ×10^9^/l | 711 (1.0) | 180 (123) | 180 (123) | 179 (123) | 186 (126) |

RCA: regional citrate anticoagulation; SHA: Systemic heparin anticoagulation; N=69,0001.

Supplementary Table 5 Sensitivity analyses and lifetime extrapolation of costs of benefits

| **Outcome** | **SHA trend, adjusted OR or difference (Δ) per year (95% CI)** | **RCA trend, adjusted OR or difference (Δ) per year (95% CI)** | **Change in trend, adjusted OR or difference (Δ) per year (95% CI)** | **Step-change, adjusted OR or difference (Δ) (95% CI)** | **Joint *p* value** |
| --- | --- | --- | --- | --- | --- |
| **Sensitivity analysis (i) – restricted to ICUs that transitioned (N=22,582)** | | | | | |
| *Primary outcome:* |  |  |  |  |  |
| 90-day mortality, OR | 1.00 (0.98–1.03) | 1.00 (0.95–1.05) | 0.99 (0.94–1.05) | 1.00 (0.89–1.13) | 0.97 |
| *Secondary outcomes:* |  |  |  |  |  |
| Duration of organ support, calendar days, Δ |  |  |  |  |  |
| Kidney support | 0.12 (0.07–0.18) | -0.09 (-0.22–0.03) | -0.22 (-0.35–-0.08) | 0.53 (0.22–0.83) | 0.00036 |
| Advanced cardiovascular support | -0.02 (-0.05–0.01) | -0.11 (-0.18–-0.05) | -0.09 (-0.16–-0.02) | 0.05 (-0.11–0.21) | 0.031 |
| Advanced respiratory support | 0.02 (-0.08–0.13) | -0.24 (-0.49–0.01) | -0.26 (-0.54–0.01) | 0.31 (-0.32–0.94) | 0.16 |
| Lengths of stay, calendar days, Δ |  |  |  |  |  |
| ICU LOS | 0.23 (0.10–0.36) | -0.26 (-0.57–0.05) | -0.49 (-0.82–-0.15) | 0.49 (-0.29–1.26) | 0.017 |
| Subsequent hospital LOS | -0.30 (-0.56–-0.04) | -0.29 (-0.90–0.32) | 0.01 (-0.65–0.67) | 0.27 (-1.24–1.79) | 0.93 |
| Total hospital LOS | -0.07 (-0.38–0.24) | -0.55 (-1.27–0.18) | -0.47 (-1.26–0.31) | 0.76 (-1.05–2.57) | 0.45 |
| Adverse events, OR |  |  |  |  |  |
| Bleeding episodes in ICU | 1.08 (1.04–1.12) | 0.97 (0.88–1.06) | 0.90 (0.81–0.99) | 0.87 (0.71–1.08) | 0.01 |
| Thromboembolic episodes up to 90 days post-discharge | 1.05 (1.01–1.10) | 1.01 (0.92–1.12) | 0.96 (0.87–1.07) | 0.86 (0.68–1.09) | 0.23 |
| ESKD treated by KRT at 90 days ^a^ | 0.99 (0.93–1.06) | 0.94 (0.79–1.12) | 0.95 (0.79–1.14) | 1.28 (0.86–1.89) | 0.47 |
| ESKD treated by KRT at one year ^b^ | 1.03 (0.97–1.09) | 0.97 (0.77–1.21) | 0.94 (0.74–1.19) | 1.12 (0.75–1.66) | 0.83 |
| Mortality, OR |  |  |  |  |  |
| Acute hospital mortality | 0.99 (0.97–1.01) | 0.99 (0.94–1.04) | 1.00 (0.95–1.06) | 1.03 (0.91–1.16) | 0.88 |
| 30–day mortality | 1.00 (0.98–1.03) | 1.02 (0.96–1.07) | 1.01 (0.96–1.07) | 0.98 (0.87–1.10) | 0.90 |
| one-year mortality | 1.01 (0.99–1.03) | 0.99 (0.94–1.04) | 0.98 (0.93–1.04) | 0.97 (0.86–1.09) | 0.59 |
| *One-year health economic outcomes* |  |  |  |  |  |
| Costs, £*,* Δ | 431 (116–747) | −835 (−1574–−96) | −1266 (−2068–−465) | 1759 (−84–3602) | 0.0058 |
| QALYs*,* Δ | −0.001 (−0.003–0.001) | 0.002 (−0.002–0.006) | 0.003 (−0.002–0.007) | 0.002 (−0.008–0.012) | 0.33 |
| INB ^c^, £*,* Δ | −451 (−768–−133) | 871 (128–1614) | 1321 (515–2128) | −1717 (−3572–137) |  |
| **Sensitivity analysis (ii) – best case costs ^c^, one-year health economic outcomes (N=69,001)** | | | | | |
| Costs, £*,* Δ | −57 (−195–81) | −689 (−1347–−30) | −632 (−1302–38) | 1615 (150–3081) | 0.073 |
| INB, £*,* Δ | 48 (−91–187) | 684 (22–1347) | 636 (−38–1310) | −1535 (−3010–−61) |  |
| **Sensitivity analysis (iii) – worst case costs ^d^, one-year health economic outcomes (N=69,001)** | | | | | |
| Costs, £*,* Δ | −66 (−202–70) | −703 (−1353–−53) | −637 (−1298–24) | 3236 (1789–4683) | 0.000051 |
| INB ^c^, £*,* Δ | 58 (−80–195) | 699 (45–1353) | 641 (−24–1307) | −3156 (−4612–−1700) |  |
| **Extrapolation to lifetime of health economic outcomes** |  |  |  |  |  |
| Costs, £*,* Δ | 533 (127–938) | −878 (−2761–1004) | −1411 (−3333–510) | 4714 (496–8933) | 0.09 |
| QALYs*,* Δ | −0.004 (−0.041–0.032) | 0.024 (−0.146–0.195) | 0.028 (−0.145–0.202) | 0.199 (−0.182–0.581) | 0.30 |
| INB ^c^, £*,* Δ | −617 (−1455–221) | 1364 (−2528–5256) | 1981 (−1990–5951) | −724 (−9446–7997) |  |

CI: confidence interval; ESKD: end-stage kidney disease; ICU: Intensive Care Unit; IQR: interquartile range; LOS: length of stay; OR: odds ratio; QALY: quality-adjusted life year; RCA: regional citrate anticoagulation; KRT: kidney replacement therapy; SHA: systemic heparin anticoagulation. ^a^ Excluding 2,497 patients with incomplete follow-up data. ^b^ Excluding 10,060 patients with incomplete follow-up data. ^c^ Lowest cost across citrate users and highest cost across heparin users from micro-costing exercise. ^d^ Highest cost across citrate users and lowest cost across heparin users from micro-costing exercise.

Supplementary Table 6 Subgroup analysis (patients meeting Sepsis-3 criteria)

| **Outcome** | **SHA trend, adjusted OR or difference (Δ) per year (95% CI)** | **RCA trend, adjusted OR or difference (Δ) per year (95% CI)** | **Change in trend, adjusted OR or difference (Δ) per year (95% CI)** | **Step-change, adjusted OR or difference (Δ) (95% CI)** | **Joint *p* value** |
| --- | --- | --- | --- | --- | --- |
| *Primary outcome:* |  |  |  |  |  |
| 90-day mortality, OR | 0.99 (0.98–1.00) | 0.98 (0.92–1.05) | 0.99 (0.93–1.06) | 1.05 (0.91–1.20) | 0.78 |
| *Secondary outcomes:* |  |  |  |  |  |
| Duration of organ support, calendar days, Δ |  |  |  |  |  |
| Kidney support | 0.09 (0.05–0.12) | −0.10 (−0.27–0.07) | −0.19 (-0.36–-0.02) | 0.56 (0.19–0.94) | 0.011 |
| Advanced cardiovascular support | −0.08 (−0.10–−0.06) | −0.23 (−0.33–−0.14) | −0.15 (-0.25–-0.05) | 0.23 (0.02–0.44) | 0.0095 |
| Advanced respiratory support | −0.06 (−0.13–0.00) | −0.41 (−0.73–−0.09) | −0.35 (-0.67–-0.02) | 0.43 (−0.28–1.13) | 0.11 |
| Lengths of stay, calendar days, Δ |  |  |  |  |  |
| ICU LOS | 0.10 (0.01–0.18) | −0.46 (−0.86–−0.06) | −0.55 (-0.96–-0.14) | 0.90 (0.01–1.79) | 0.026 |
| Subsequent hospital LOS | −0.25 (−0.41–−0.10) | −0.47 (−1.19–0.25) | −0.22 (-0.95–0.52) | 0.43 (−1.19–2.06) | 0.83 |
| Total hospital LOS | −0.16 (−0.36–0.03) | -0.96 (−1.88–−0.03) | −0.80 (-1.74–0.15) | 1.20 (−0.87–3.27) | 0.25 |
| Adverse events, OR |  |  |  |  |  |
| Bleeding episodes in ICU | 1.06 (1.04–1.09) | 0.99 (0.88–1.11) | 0.93 (0.82–1.05) | 0.90 (0.69–1.16) | 0.069 |
| Thromboembolic episodes up to 90 days post-discharge | 1.04 (1.02–1.07) | 0.99 (0.87–1.13) | 0.95 (0.83–1.08) | 0.90 (0.68–1.19) | 0.22 |
| ESKD treated by KRT at 90 days ^a^ | 1.06 (1.00–1.12) | 1.13 (0.88–1.45) | 1.07 (0.83–1.38) | 0.89 (0.51–1.56) | 0.86 |
| ESKD treated by KRT at one year ^b^ | 1.11 (1.05–1.18) | 1.02 (0.74–1.41) | 0.92 (0.66–1.27) | 0.76 (0.43–1.36) | 0.23 |
| Mortality, OR |  |  |  |  |  |
| Acute hospital mortality | 0.98 (0.97–0.99) | 0.97 (0.91–1.03) | 0.99 (0.93–1.05) | 1.12 (0.98–1.28) | 0.22 |
| 30–day mortality | 0.99 (0.98–1.01) | 1.00 (0.94–1.06) | 1.00 (0.94–1.07) | 1.04 (0.91–1.19) | 0.78 |
| one-year mortality | 0.99 (0.98–1.00) | 0.97 (0.92–1.04) | 0.98 (0.92–1.05) | 1.06 (0.92–1.22) | 0.70 |
| *One-year health economic outcomes* |  |  |  |  |  |
| Costs, £*,* Δ | 109 (−90–307) | −1067 (−2016–−119) | −1176 (−2142–−210) | 2633 (531–4735) | 0.025 |
| QALYs*,* Δ | 0.001 (−0.000–0.002) | 0.002 (−0.003–0.007) | 0.001 (−0.004–0.007) | −0.005 (−0.016–0.007) | 0.74 |
| INB ^c^, £*,* Δ | −93 (−293–107) | 1112 (158–2066) | 1205 (234–2176) | −2725 (−4840–−610) |  |

CI: confidence interval; ESKD: end-stage kidney disease; ICU: Intensive Care Unit; IQR: interquartile range; LOS: length of stay; OR: odds ratio; QALY: quality-adjusted life year; RCA: regional citrate anticoagulation; KRT: kidney replacement therapy; SHA: systemic heparin anticoagulation. N=32,772. ^a^ Excluding 2,497 patients with incomplete follow-up data. ^b^ Excluding 10,060 patients with incomplete follow-up data.

# Supplementary Material references

[1] Curtis L, Burns A. Unit Costs of Health and Social Care 2018. 2019. Canterbury, UK: Personal Social Services Research Unit, University of Kent; 2018.

[2] NHS Business Services Authority. NHS Business Serivces Authority Drug Tariff. NHS Business Services Authority; 2019.

[3] Chain NS. NHS Supply Chain, <https://www.supplychain.nhs.uk/>; 2019 [accessed 29 October.2019].

[4] Bedford JP, Gerry S, Hatch RA, Rechner I, Young JD, Watkinson PJ. Hospital outcomes associated with new-onset atrial fibrillation during ICU admission: A multicentre competing risks analysis. J Crit Care 2020;60:72-8.

[5] Marshall DC, Hatch RA, Gerry S, Young JD, Watkinson P. Conditional Survival With Increasing Duration of ICU Admission: An Observational Study of Three Intensive Care Databases. Crit Care Med 2020;48(1):91-7.

[6] Lefrant JY, Garrigues B, Pribil C, Bardoulat I, Courtial F, Maurel F, et al. The daily cost of ICU patients: A micro-costing study in 23 French Intensive Care Units. Anaesthesia, critical care & pain medicine 2015;34(3):151-7.

[7] Department of Health. Department of Health. NHS reference costs, <https://www.gov.uk/government/collections/nhs-reference-costs>; 2016 [accessed 03 Oct 2017.

[8] Hatch R, Young D, Barber V, Harrison DA, Watkinson P. The effect of postal questionnaire burden on response rate and answer patterns following admission to intensive care: a randomised controlled trial. BMC Med Res Methodol 2017;17(1):49.

[9] Eriksson D, Goldsmith D, Teitsson S, Jackson J, van Nooten F. Cross-sectional survey in CKD patients across Europe describing the association between quality of life and anaemia. BMC Nephrol 2016;17(1):97.

[10] Dolan P, Gudex C, Kind P, Williams A. A Social Tariff for EuroQoL: Results from a UK General Population Survey. CHE Discussion Paper*.* York: Centre for Health Economics: University of York; 1999.

[11] Wright JC, Plenderleith L, Ridley SA. Long-term survival following intensive care: subgroup analysis and comparison with the general population. Anaesthesia 2003;58(7):637-42.

[12] Cuthbertson BH, Roughton S, Jenkinson D, Maclennan G, Vale L. Quality of life in the five years after intensive care: a cohort study. Crit Care 2010;14(1):R6.

[13] Dowdy DW, Eid MP, Sedrakyan A, Mendez-Tellez PA, Pronovost PJ, Herridge MS, et al. Quality of life in adult survivors of critical illness: a systematic review of the literature. Intensive Care Med 2005;31(5):611-20.

[14] Ara R, Brazier JE. Populating an economic model with health state utility values: moving toward better practice. Value Health 2010;13(5):509-18.
